# Arthrocentesis approaches to the phalangeal joints of the one humped camel (*Camelus dromedarius*)

**DOI:** 10.1038/s41598-023-44391-1

**Published:** 2023-10-13

**Authors:** Ahmad Al Aiyan, Fransina Christina King, Adnan Aldarwich, Uday Kishore, Turke Shawaf

**Affiliations:** 1 Department of Veterinary Medicine, College of Agriculture and Veterinary Medicine, United Arab Emirates University, Al Ain, UAE; 2 Department of Clinical Sciences, College of Veterinary Medicine, King Faisal University, Al-Hasa, Saudi Arabia

**Keywords:** Zoology, Anatomy

## Abstract

Irrespective of the exceptional adaptation of dromedaries to harsh environmental conditions, they remain highly susceptible to joint lameness resulting from a range of diverse factors and conditions. The joints most often affected by traumatic osteoarthritis in dromedaries are the metacarpophalangeal and metatarsophalangeal joints. A comprehensive understanding of joint anatomy and topography of the dromedary is required to perform arthrocentesis correctly on affected joints. Forty-two distal limbs were taken from 28 camels and studied by gross dissection, casting, ultrasonography, and computed tomography (CT). Representative three-dimensional models of the joint cavities, recesses, and pouches were obtained using different casting agents. This study provides a detailed description of dorsally, axially, and abaxially positioned joint recesses, as well as palmar/plantar positioned joint pouches. The safety and feasibility of the different arthrocentesis approaches were evaluated. The traditional dorsal arthrocentesis approach of the metacarpophalangeal, metatarsophalangeal, proximal interphalangeal, and distal interphalangeal joints, has limitations due to the risk of damaging the tendon structures and articular cartilage, which can lead to joint degeneration. A lateral arthrocentesis approach via the proximal palmar/plantar pouches of the metacarpophalangeal/metatarsophalangeal and proximal interphalangeal joints is recommended. This approach eliminates the potential needle injury to the articulating joint cartilage and other surrounding joint structures, such as tendons, blood vessels, and nerves.

## Introduction

Dromedaries contribute considerably to livestock production in the Middle East^1–3^. Their biomechanical development enables them to be exceptionally well-adjusted to the desert environment^4–8^, in which they thrive as valuable racing, breeding, and showing animals^2,4,6^. Irrespectively dromedaries remain highly susceptible to joint lameness resulting from conditions such as osteoarthritis, immune-mediated polyarthritis, and infectious synovitis^4,9,10^.

Undiagnosed and untreated joint disorders are detrimental to the wellbeing, production potential, and life expectancy of dromedaries^9,10^. Untreated synovitis leads to complications, including cartilage and subsequent bone degradation^11–13^. The associated costs of care and treatment, poor physical performance, production loss, and early retirement of valuable animals contribute to the economic impact caused by lameness^4,9^. Lameness related to joint diseases has undeniably strong clinical importance and required an accurate technique to perform joint arthrocentesis in practice^6,14,15^.

Some of the joints most affected by traumatic arthritis in dromedaries are the high-motion metacarpophalangeal and metatarsophalangeal joints^9^. These joints have a unique function in dromedaries. When dromedaries stand up, these joints bear considerable stress and weight in excessively flexed positions.

Limited research has been published about the detailed anatomy and topography of the joint structures in dromedaries. However, a significant number of publications on equine and bovine joint anatomy, lameness, joint health, diagnostics, and treatment have been published^2,16,17^. However, the distinct anatomical structure of the phalangeal joints of dromedaries^5–8,15,18^ presents a challenge when comparing the patterns of lameness and arthrocentesis techniques commonly observed and utilized in bovines and equines to those employed in camels. This comparison is essential to understand the unique aspects of lameness in dromedaries, particularly before and after intra-articular blocks^4,12^.

Preliminary comprehensive research on the anatomy and topography of healthy joints of the dromedary camel is required to establish the effective diagnosis and treatment of joints affected by osteoarthritis^19–22^. This essential information will assist in optimizing arthrocentesis of the phalangeal joints in dromedaries^6,19^. Accurate needle placement during arthrocentesis is importance to prevent damage to the articular cartilage surface and surrounding structures^23^. Ultrasonography can be used to successfully guide needle placement during arthrocentesis to avoid unnecessary complications^15,24^.

The investigation and diagnosis of affected joints are supported by scanning methods such as computed tomography (CT), 3D rendering, and ultrasonography^18,19,23,25^. A reference database of non-pathological images is of paramount importance to serve as a basis for comparison when viewing scans of affected joints^26^.

This study aimed to describe the basic anatomy and topography of the metacarpophalangeal/metatarsophalangeal and proximal and distal interphalangeal joints, including the synovial cavities, synovial capsule outlines, synovial recesses, and synovial pouches in non-pathological joints of the dromedary. This study also aimed to provide guidelines for ultrasonography of the phalangeal joints. Furthermore, this study aimed to re-evaluate current arthrocentesis techniques and provide detailed descriptions of arthrocentesis approaches for the phalangeal joints in the dromedary.

## Materials and methods

In addition to adhering to the Research Ethics Policy, the experimental protocol conducted in this study was approved by the Animal Research Ethics Committee at the United Arab Emirates University (ERA_2020_6088). We confirm that this study is reported in accordance with Animal Research: Reporting of in vivo Experiments (ARRIVE) guidelines. In accordance with the guidelines set forth by the United Arab Emirates University, we have followed a standardized approach to reporting our study, thereby enhancing the quality and reliability of our findings.

### Experimental design

Dromedary distal forelimbs and hindlimbs were obtained from two local abattoirs located in Abu Dhabi, UAE (Al Khazna and Al Bawadi). In total 42 distal limbs were harvested from 28 dromedaries’ carcasses post-slaughter. The distal forelimbs and hindlimbs were amputated 10 cm proximal to the carpal or tarsal joints, respectively. The samples were collected from male (n = 16) and female (n = 12) animals between 1 and 4 years old. Samples reached the laboratory within 4 h post-slaughter. The samples were immediately used as described in the methods below. Table 1 provides an indication of the number of samples collected.

**Table 1:** The total number of distal forelimbs and hindlimbs collected.

Distal limbs: n = 42	Distal forelimbs: n = 35	Metacarpophalangeal joints: n = 70
		Proximal interphalangeal joint: n = 70
		Distal interphalangeal joint: n = 70
	Distal hindlimbs: n = 7	Metatarsophalangeal joints: n = 14
		Proximal interphalangeal joint: n = 14
		Distal interphalangeal joint: n = 14

### Arthrocentesis

A dorsal and lateral arthrocentesis approach was used to cast the various phalangeal joints. Table 2 indicates the total number of samples injected from a dorsal or lateral approach.

**Table 2:** The arthrocentesis approaches per sample type.

Dorsal approach n = 32	Distal forelimbs: n = 28	Metacarpophalangeal joints: n = 56
		Proximal interphalangeal joint: n = 56
		Distal interphalangeal joint: n = 56
	Distal hindlimbs: n = 4	Metatarsophalangeal joints: n = 8
		Proximal interphalangeal joint: n = 8
		Distal interphalangeal joint: n = 8
Lateral approach 10	Distal forelimbs: n = 7	Metacarpophalangeal joints: n = 14
		Proximal interphalangeal joint: n = 14
	Distal hindlimbs: n = 3	Metatarsophalangeal joints: n = 6
		Proximal interphalangeal joint: n = 6

The equipment used to perform the arthrocentesis was precisely duplicated from that which is described in King et al.^15^.

**Figure 1:**
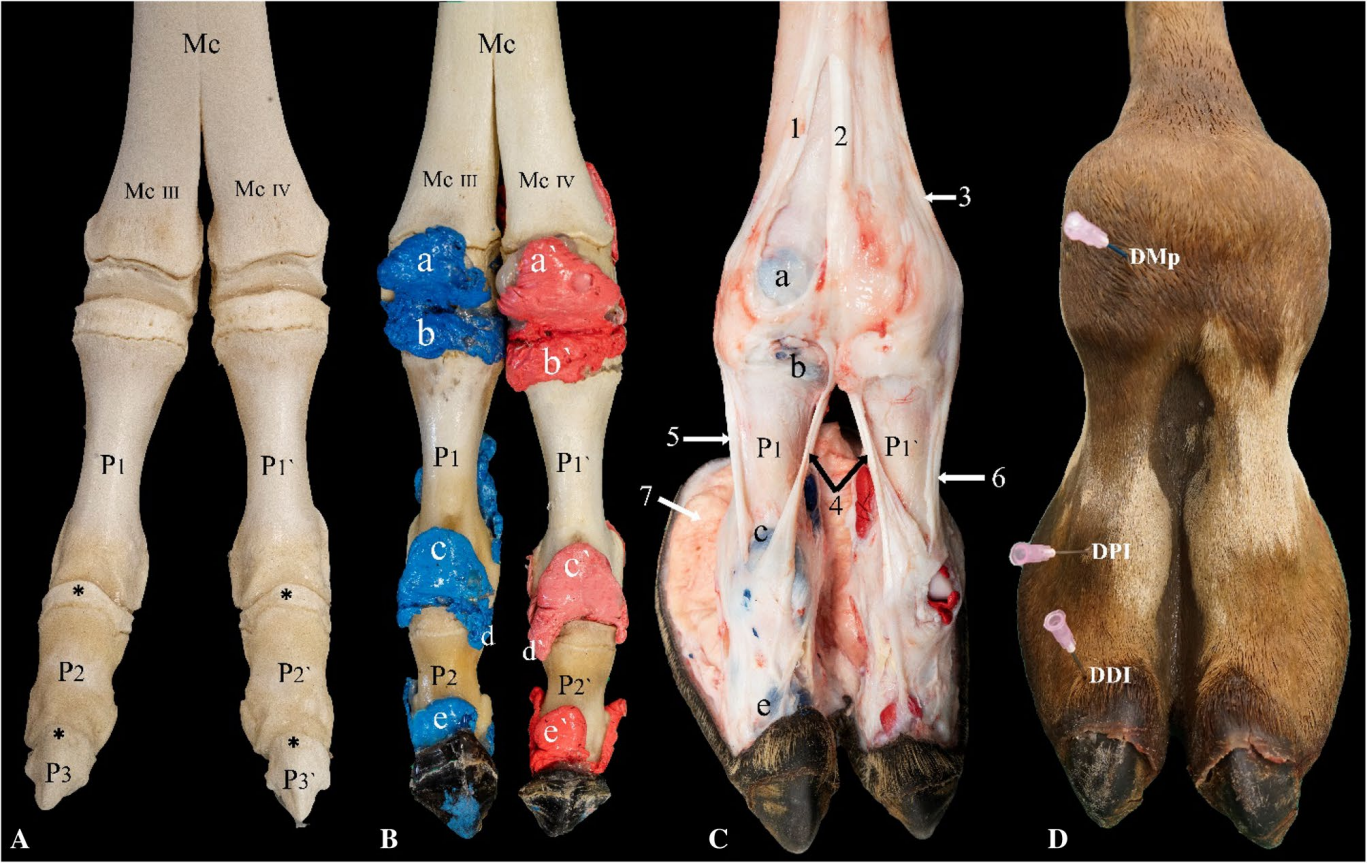
Dorsal view of the left distal forelimb. (**A**) Bones, (**B**) casting, (**C**) dissection, and (**D**) arthrocentesis approaches on an intact specimen. *Mc* metacarpal bone, *Mc III* distal end of metacarpal bone III, *Mc IV *distal end of metacarpal bone IV, *P1 and P1′* proximal phalanx, *P2 and P2′* middle phalanx, *P3 and P3′* distal phalanx, *a and a′* proximal dorsal recess of the metacarpophalangeal joint, *b and b′* distal dorsal recess of the metacarpophalangeal joint, *c and c′* proximal dorsal recess of the proximal interphalangeal joint, *d and d’* distal axial recess of the proximal interphalangeal joint, *e and e′* proximal dorsal recess of the distal interphalangeal joint, *1* medial tendon of the common digital extensor muscle, *2* lateral tendon of the common digital extensor muscle, *3* lateral digital extensor tendon, *4* continuation of the lateral tendon of the common digital extensor muscle, *5* proper extensor tendon of digit III, *6* proper extensor tendon of digit IV, *7* fat pad, *DMp* dorsal arthrocentesis approach to the metacarpophalangeal joint, *DPI* dorsal arthrocentesis approach to the proximal interphalangeal joint, *DDI* dorsal arthrocentesis approach to the distal interphalangeal joint. The black asterisks indicate the extensor process of the middle and distal phalanx.

When using the dorsal approach to the metacarpophalangeal/metatarsophalangeal joints, the joints were flexed, and the depression formed between the distal end of the metacarpal/metatarsal bone and the proximal end of the proximal phalanx (P1) was palpated. A 14-gauge needle attached to a closed three-way-stop-cock catheter was inserted at the center of the depression (Fig. 1D). The proximal and distal interphalangeal joints were also flexed when using the dorsal approach; the 14-gauge needle was inserted lateral to the extensor process of the middle phalanx (P2) and distal phalanx (P3), respectively (Fig. 1A,D).

When using the lateral approach, the 14-gauge needle was inserted into the proximal palmar/plantar pouch of the metacarpophalangeal/metatarsophalangeal and proximal interphalangeal joints under high-frequency ultrasound guidance or via palpation approach (Fig. 4C,D). The ultrasound guidance was performed with a MyLabOmegaVet (series 7400 model) (Esaote, Florence, Italy) ultrasound device in combination with an Esaote L4–15 (4–15 MHz; 47 mm) linear transducer (Esaote, Florence, Italy). When using the palpation approach, the point of needle insertion for the proximal palmar/plantar pouch of the metacarpophalangeal/metatarsophalangeal joint was identified where the depression was formed proximal to the abaxial proximal sesamoid bone, the interosseous medius muscle (IOM), and the metacarpal/metatarsal bone. The injection was performed immediately dorsal to the tendon of the IOM before its insertion to the abaxial proximal sesamoid bone (Fig. 4C,D). At this level, the 14-gauge needle was inserted at a 45° angle and directed toward the distal part of the metacarpal/metatarsal bone. The point of needle insertion to the proximal palmar/plantar pouch of the proximal interphalangeal joint was identified where a depression between the abaxial articular prominence of P1 and the superficial digital flexor (SDF) tendon was formed (Fig. 4C,D). The 14-gauge needle was inserted dorsal to the tendon of the SDF at a 45° angle directed toward the distal end of P1.

### Casting

**Figure 2:**
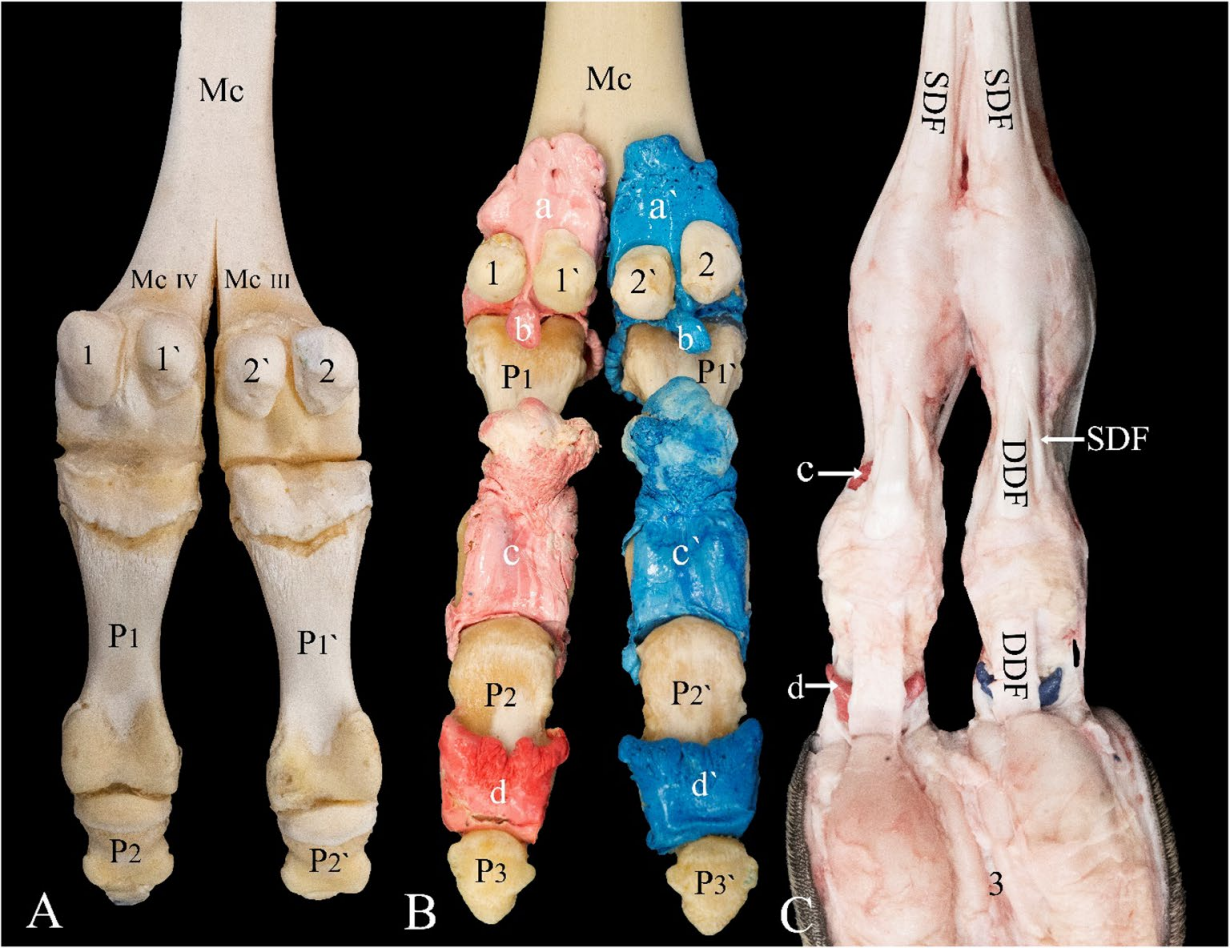
Palmar view of the left distal forelimb. (**A**) Bones, (**B**) casting, and (**C**) dissection. *Mc* metacarpal bone, *Mc III* distal end of metacarpal bone III, *Mc IV* distal end of metacarpal bone IV, *P1 and P1′* proximal phalanx, *P2 and P2′* middle phalanx, *P3 and P3′* distal phalanx, *1 and 1′* abaxial and axial proximal sesamoid bone of digit IV, *2 and 2′* abaxial and axial proximal sesamoid bone of digit III, *3* fat pad, *a and a′* proximal palmar pouch of the metacarpophalangeal joint, *b and b′* distal palmar pouch of the metacarpophalangeal joint, *c and c′* proximal palmar pouch of the proximal interphalangeal joint, *d and d′* proximal palmar pouch of the distal interphalangeal joint, *SDF* superficial digital flexor tendon, *DDF* deep digital flexor tendon.

EasyFlo 60 Liquid Plastic (Polytek Development Corp, Easton, PA, USA) was used to cast samples. The preparation of the casting agent followed the guidelines and techniques specified by Al Aiyan et al.^27,28^. Blue and red dye were used alternatively to color the casting agents to effortlessly distinguish between joint cavities of digits III and IV of each distal limb (Figs. 1B, 2B, 4B).

Synovial fluid was extracted from the joint cavities before the respective casting material was injected. The casting material was injected gradually with manual pressure using a 20 ml syringe until resistance was felt. The 14-gauge needle and catheter were not removed to prevent any backflow of the casting material. After casting, the samples were kept at room temperature for 4 h and then moved into a cold room at a temperature of 5 °C. Samples were held for a minimum of 24 h before dissection or 48 h before maceration.

### Maceration

The surrounding soft tissue was removed from the 35 distal limbs in preparation for maceration. Samples were placed in a 2% sodium bicarbonate and water (w/v) solution and incubated at 40 °C for 48 h. The samples were removed, and the 2% sodium bicarbonate and water (w/v) solution were discarded. The saturated soft tissue was manually removed, and the samples were placed into a freshly mixed 2% sodium bicarbonate and water (w/v) solution and incubated at 40 °C for 72 h. The bone specimens were removed and allowed to dry for 24 h. Any needed gluing or refixing of structures to the samples was performed accordingly by a lab specialist.

### Dissection

**Figure 3:**
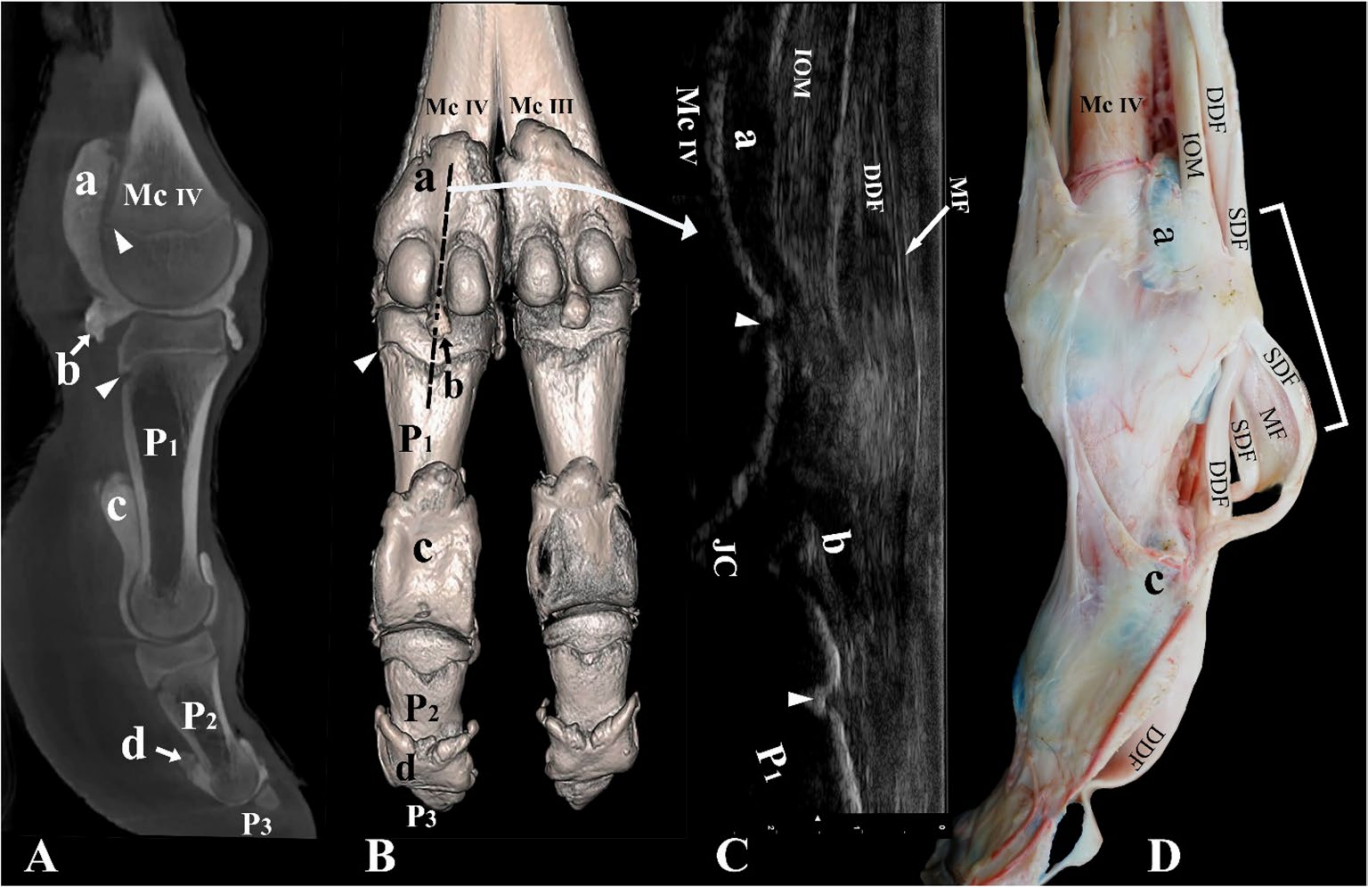
Palmar and lateral view of the left distal forelimb and digit IV. (**A**) Sagittal plane CT image, (**B**) three-dimensional reconstruction (the black dotted line indicates the placement of the ultrasound probe between the two proximal sesamoid bones), (**C**) longitudinal palmar ultrasound image of the metacarpophalangeal joint, and (**D**) dissection. *Mc III* distal end of metacarpal bone III, *Mc IV* distal end of metacarpal bone IV, *P1* proximal phalanx, *P2* middle phalanx, *P3* distal phalanx, *a* proximal palmar pouch of the metacarpophalangeal joint, *b* distal palmar pouch of the metacarpophalangeal joint, *c* proximal palmar pouch of the proximal interphalangeal joint, *d* proximal palmar pouch of the distal interphalangeal joint, *IOM* interosseous medius muscle, *SDF* superficial digital flexor tendon, *DDF* deep digital flexor tendon, *MF* manica flexoria, *JC* joint cavity. The white arrowheads indicate the epiphyseal lines, and the white square bracket indicates the ultrasound area on image (**D**).

**Figure 4:**
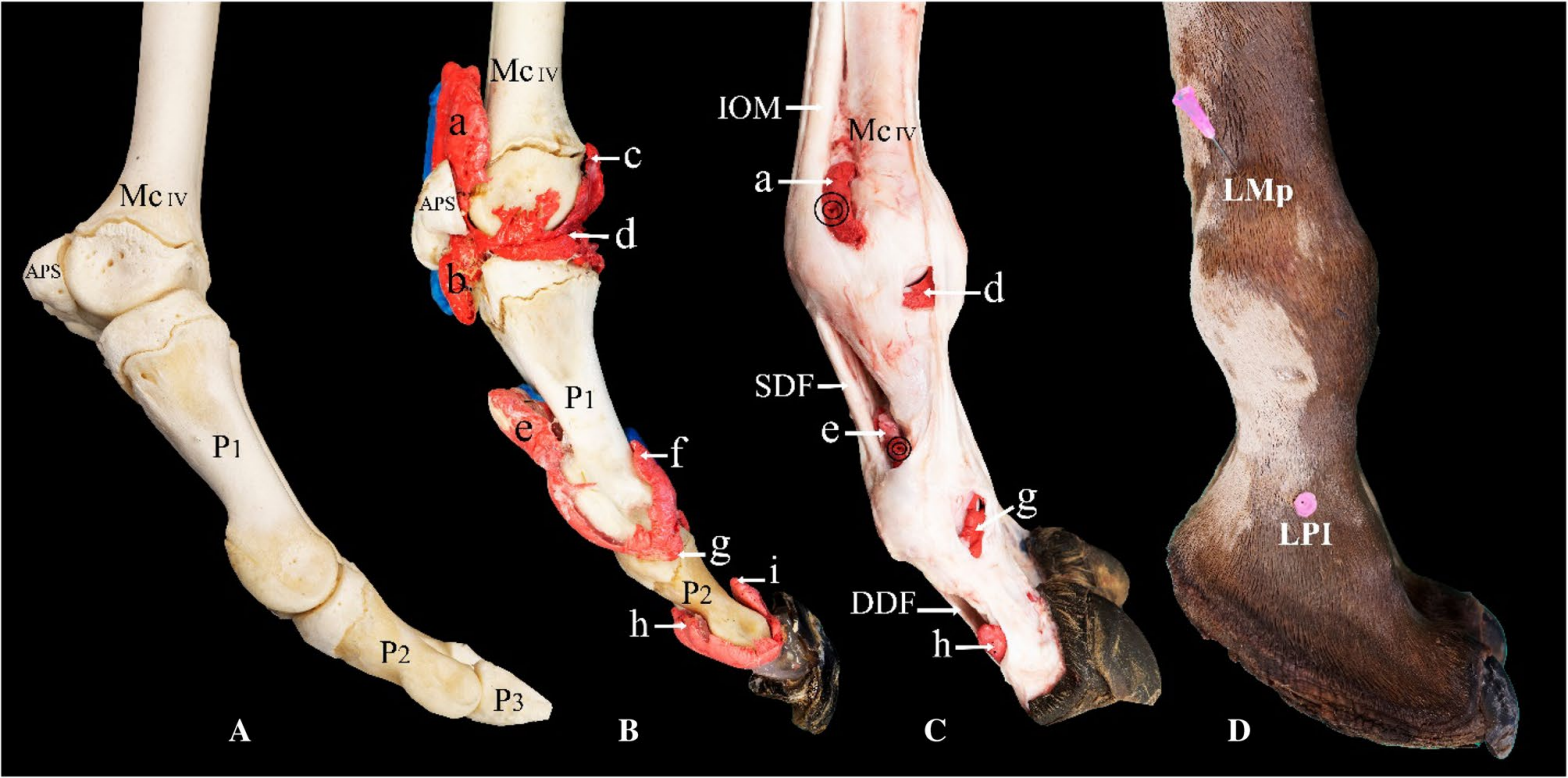
Lateral view of the left distal forelimb. (**A**) Bones, (**B**) casting, (**C**) dissection, and (**D**) arthrocentesis approaches on an intact specimen. *Mc IV* distal end of metacarpal bone IV, *P1* proximal phalanx, *P2* middle phalanx, *P3* distal phalanx, *APS* abaxial proximal sesamoid bone, *a* proximal palmar pouch of the metacarpophalangeal joint, *b* distal palmar pouch of the metacarpophalangeal joint, *c* proximal dorsal recess of the metacarpophalangeal joint, *d* metacarpophalangeal joint cavity, *e* proximal palmar pouch of the proximal interphalangeal joint, *f* proximal dorsal recess of the proximal interphalangeal joint, *g* distal abaxial recess of the proximal interphalangeal joint, *h* proximal palmar pouch of the distal interphalangeal joint, *i* proximal dorsal recess of the distal interphalangeal joint, *IOM* interosseous medius muscle, *SDF* superficial digital flexor tendon, *DDF* deep digital flexor tendon, *LMp* lateral arthrocentesis approach of the proximal palmar pouch of the metacarpophalangeal joint, *LPI* lateral arthrocentesis approach of the proximal palmar pouch of the interphalangeal joint. Double encircled areas indicate the needle insertion point via a lateral injection approach.

All casted distal limbs were dissected to examine the impact of the needle on surrounding joint structures during arthrocentesis. The potential joint cavity communications with surrounding tendon sheaths, joint capsules, and joint recesses and pouches were also extensively examined (Figs. 1C, 2C, 3D, 4C). The gross anatomy of the space between the articulating bone surfaces, the range of joint flexion, and the exposure of the joint cartilage surface during flexion was also examined.

### Ultrasonography

**Figure 5:**
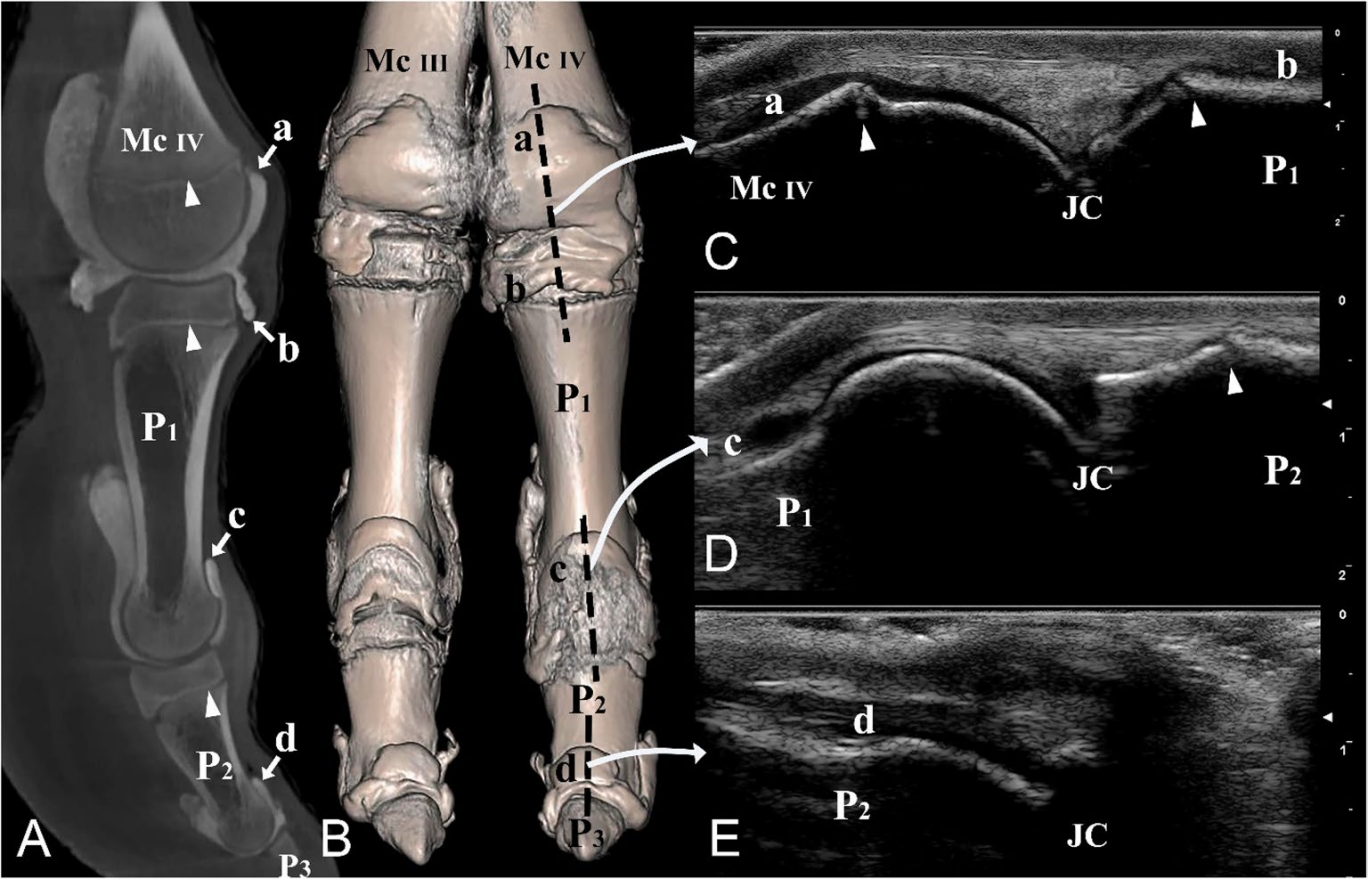
Dorsal and lateral views of the left distal forelimb and digit IV. (**A**) Sagittal plane CT image; (**B**) three-dimensional reconstruction (the black dotted line indicating the dorsal placement of the ultrasound probe), (**C**) longitudinal dorsal ultrasound image of the metacarpophalangeal joint, (**D**) longitudinal dorsal ultrasound image of the proximal interphalangeal joint, and (**E**) longitudinal dorsal ultrasound image of the distal interphalangeal joint. *Mc III* distal end of metacarpal bone III, *Mc IV* distal end of metacarpal bone IV, *P1* proximal phalanx, *P2* middle phalanx, *P3* distal phalanx, *a* proximal dorsal recess of the metacarpophalangeal joint, *b* distal dorsal recess of the metacarpophalangeal joint, *c* proximal dorsal recess of the proximal interphalangeal joint, *d* proximal dorsal recess of the distal interphalangeal joint, *JC* joint cavity. White arrowheads indicate the epiphyseal lines.

**Figure 6:**
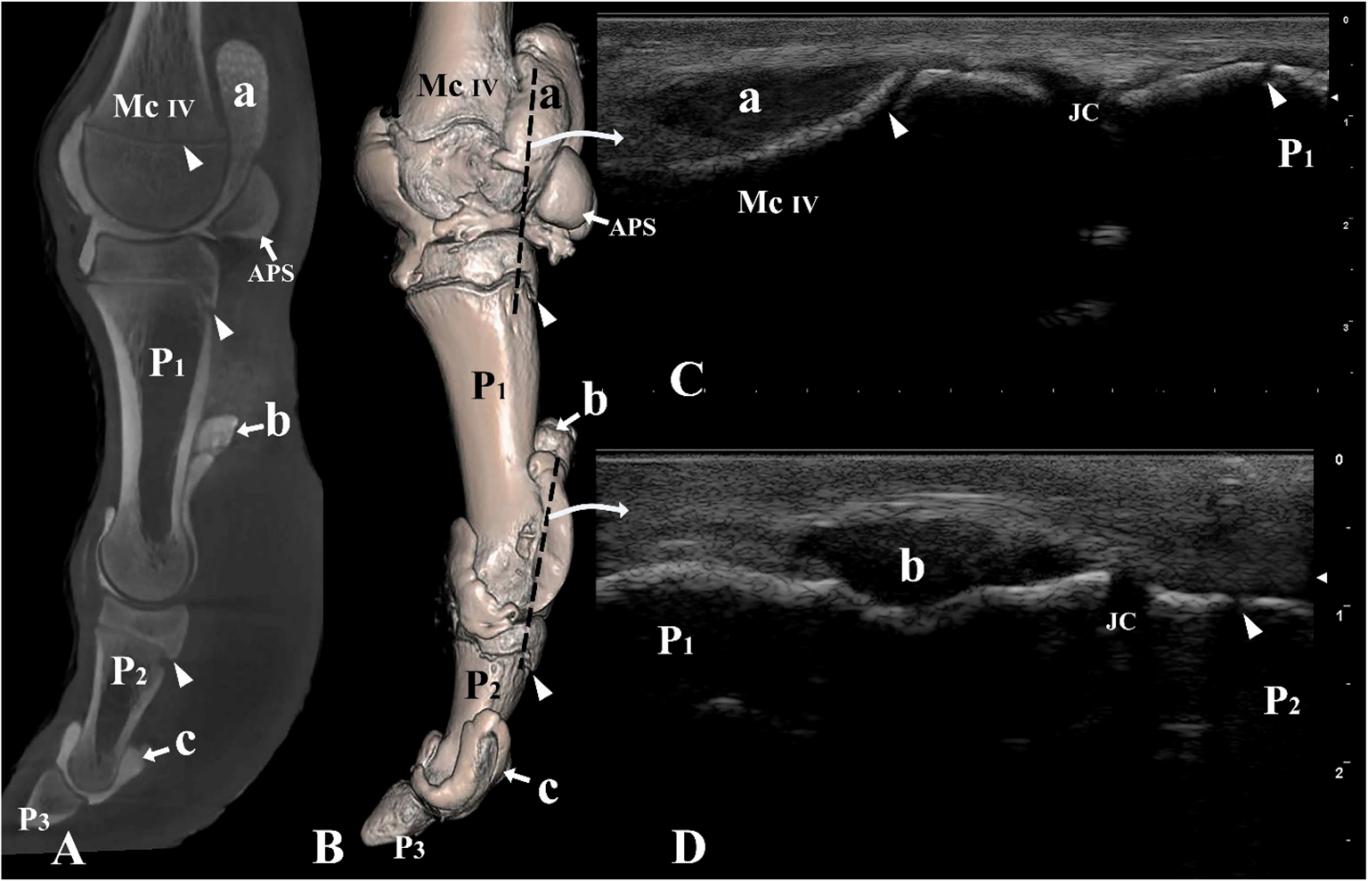
Lateral view of the left digit IV. (**A**) Sagittal plane CT image; (**B**) three-dimensional reconstruction (the black dotted line indicating the placement of the ultrasound probe), (**C**) longitudinal lateral ultrasound image of the metacarpophalangeal joint, and (**D**) longitudinal lateral ultrasound image of the proximal interphalangeal joint. *Mc IV* distal end of metacarpal bone IV, *P1* proximal phalanx, *P2* middle phalanx, *P3* distal phalanx, *APS* abaxial proximal sesamoid bone, *a* proximal palmar pouch of the metacarpophalangeal joint, *b* proximal palmar pouch of the proximal interphalangeal joint, *c* proximal palmar pouch of the distal interphalangeal joint, *JC* joint cavity. White arrowheads indicate the epiphyseal lines.

**Figure 7:**
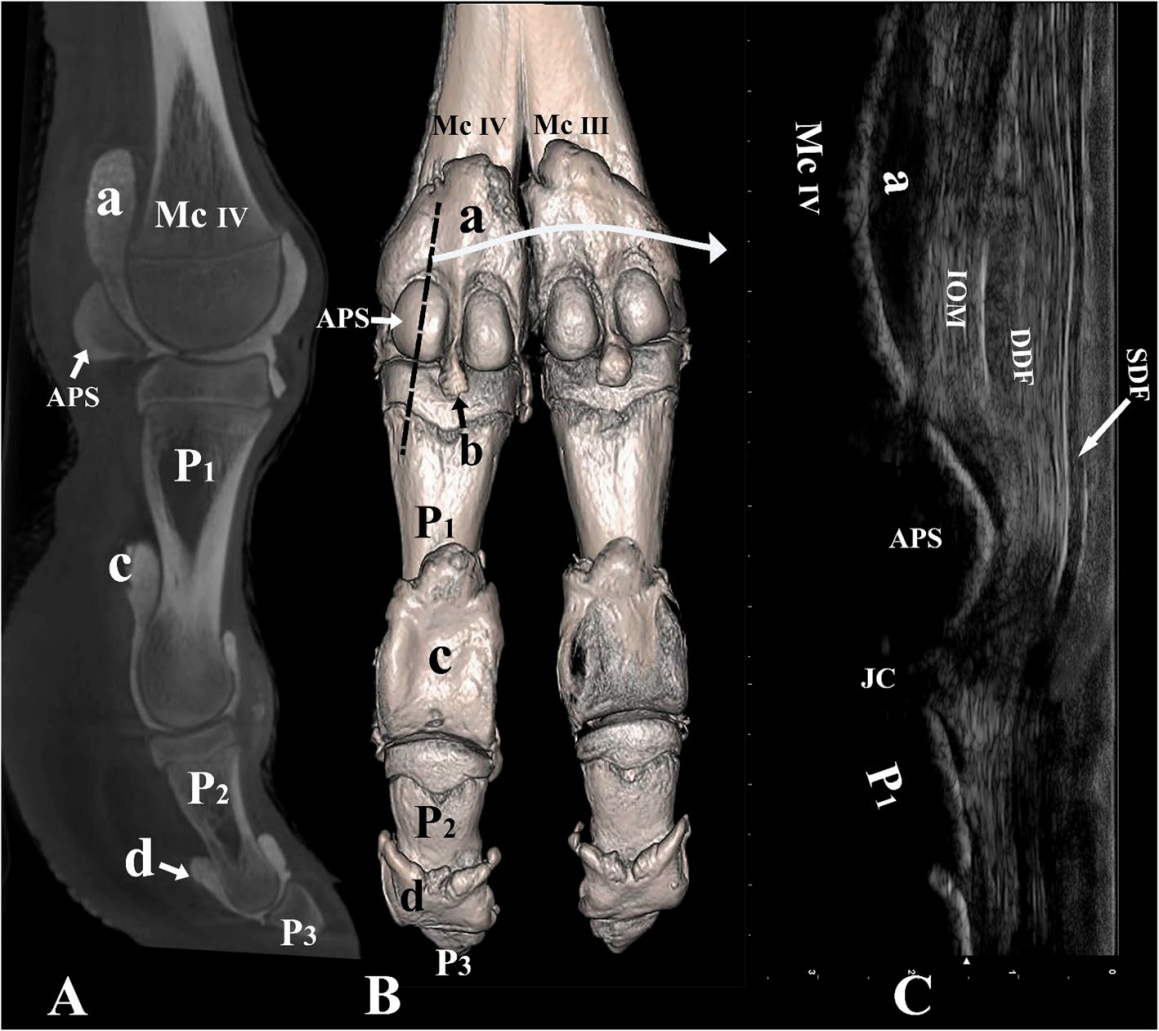
Palmar and lateral view of the left distal forelimb and digit IV. (**A**) Sagittal plane CT image; (**B**) three-dimensional reconstruction (the black dotted line indicating the placement of the ultrasound probe over the abaxial proximal sesamoid bone), and (**C**) longitudinal palmar ultrasound image of the metacarpophalangeal joint. *Mc III* distal end of metacarpal bone III, *Mc IV* distal end of metacarpal bone IV, *P1* proximal phalanx, *P2* middle phalanx, *P3* distal phalanx, *APS* abaxial proximal sesamoid bone, *a* proximal palmar pouch of the metacarpophalangeal joint, *b* distal palmar pouch of the metacarpophalangeal joint, *c* proximal palmar pouch of the proximal interphalangeal joint, *d* proximal palmar pouch of the distal interphalangeal joint, *IOM* interosseous medius muscle, *SDF* superficial digital flexor tendon, *DDF* deep digital flexor tendon, *JC* joint cavity.

Twenty digits were examined using ultrasonography. A MyLabOmegaVet (series 7400 model) (Esaote, Florence, Italy) ultrasound device combined with an Esaote L4-15 (4–15 MHz; 47 mm) linear transducer (Esaote, Florence, Italy) was used. The equine tendon superficial pre-setting was selected to obtain the best quality images (Figs. 3C, 5C–E, 6C,D, 7C). The digits were shaved and placed horizontally on the table and a generous amount of ultrasound gel was used.

The dorsal and lateral aspects of the digits were investigated longitudinally in an extended position. The probe was moved distally in a parallel line over the respective joints. The metacarpophalangeal and metatarsophalangeal joints were ultrasonographically investigated from a palmar/plantar aspect. During this investigation, the probe was moved distally either over the axial or abaxial proximal sesamoid bones or between the two proximal sesamoid bones. For illustrative purposes, the ultrasound images in Figs. 3C, 5C–E, 6C,D and 7C were composed by combining two individual ultrasound images through using Adobe Photoshop 2022 (Adobe, San Jose, CA, USA). Furthermore, we conducted ultrasound assessments in the dorsal, palmar, and plantar aspects of the proximal interphalangeal joints. However, access to the palmar/plantar aspect of the distal interphalangeal joint was limited due to footpad restriction.

### Computed tomography

Four metacarpophalangeal, metatarsophalangeal, proximal interphalangeal, and distal interphalangeal joints were cast with Globalsil AL 20 Flexible Molds Rubber (GLOBALCHIMICA S.r.l., Piedmont, Turin, Italy) mixed with a contrast medium called Omnipaque (350 mg/ml) (GE Healthcare, Chicago, IL, USA). CT images were taken at the Dubai Equine Hospital, using an EQUIMAGINE™ multi-modality robotic scanner (Universal Medical Systems, Inc., Solon, OH, USA). CT scanning was conducted at 100 kV and 0.50 mAs. Transverse and sagittal series were obtained at a thickness of 0.5 mm (Figs. 3A, 5A, 6A, 7A). Reconstructed three-dimensional models were derived from the CT scans using Horos™ software (OsiriX™, Los Angeles, CA, USA) (Figs. 3B, 5B, 6B, 7B).

## Results

### Anatomy and casting

The two metacarpophalangeal and two metatarsophalangeal joints are formed by the trochlea of metacarpal/metatarsal bone III and IV, the proximal articular surface of P1, and the palmar/plantar located proximal sesamoid bones (Figs. 1A, 2A, 4A). Each of these joints has an individual joint capsule, which means that the two joint cavities of the metacarpophalangeal and metatarsophalangeal joints are separated (Fig. 1B).

Dorsally, the metacarpophalangeal/metatarsophalangeal joint capsule forms a proximal recess that extends to the level of the epiphyseal line of the metacarpal/metatarsal bone (Fig. 1B). Another recess is formed by the capsule in a dorsodistal manner and extends to the level of the epiphyseal line of P1 (Fig. 1B). Palmar/plantar, the joint capsule forms a large proximal pouch that extends beyond the epiphyseal line of the metacarpal/metatarsal bone and the associated intertrochlear notch. The joint capsule also forms a short distopalmar pouch between the two sesamoid bones (Fig. 2B).

The proximal interphalangeal joint is formed by the articulation of the trochlea of P1 and the proximal articulation surface of P2. Its joint capsule forms a short proximal dorsal recess on P1 (Fig. 1B). It also forms axially and abaxially a short recess that extends distally on P2 (Figs. 1B, 4B). Furthermore, it forms a large palmar/plantar pouch that extends proximally up to two-thirds of P1 (Fig. 2B). The distal interphalangeal joint is formed between the trochlea of P2 and the proximal articulation surface of P3. The joint capsule forms a proximal dorsal recess that extends up to the distal third of P2 (Fig. 1B). It also forms a proximal palmar/plantar pouch that extends up to the midsection of the P2 (Fig. 2B).

Dorsally, the medial tendon of the common digital extensor muscle moves mediodistally over the metacarpophalangeal/metatarsophalangeal joint of digit III and partially fuses with the joint capsule and inserts on the proximal dorsal aspect of the P1. The more abaxial part of the tendon continues distally and is referred to as the proper extensor tendon of digit III. It ultimately inserts on the dorsal surface of P2 and P3 (Fig. 1C). The lateral tendon of the common digital extensor muscle bifurcates at the level between the metacarpophalangeal/metatarsophalangeal joints. The tendon inserts on the proximal dorsal aspect of P2 and distally on the extensor process of P3. At this level, the tendon is referred to as the true common extensor tendon (Fig. 1C). The lateral digital extensor tendon fuses with the dorsal surface of the metacarpophalangeal/metatarsophalangeal joint capsule of digit IV. It continues distally and is referred to as the proper extensor tendon of digit IV. It fuses with the proximal interphalangeal joint capsule and continues distally to insert on the proximal dorsal surface of P2 and ultimately on the extensor process of P3 (Fig. 1C).

Palmar/plantar, the IOM, SDF, and deep digital flexor (DDF) muscles bifurcate in the distal region of the metacarpal/metatarsal bone. The lateral and medial tendinous band of the IOM inserts on the proximal sesamoid bones of digits III and IV (Figs. 3C,D, 4C, 7C). It does not extend a dorsal supporting branch to fuse with the extensor tendons. As the SDF and DDF move over the metacarpophalangeal/metatarsophalangeal joint, the SDF gives rise to the manica flexoria (Fig. 3D). The middle part of the tendon becomes thin and the borders of the tendon remain thick as it moves over the proximal sesamoid bones (Figs. 3D, 7C). Distal to the metacarpophalangeal/metatarsophalangeal joint, the SDF forms a slit and allows the tendon of the DDF to move through the manica flexoria (Figs. 2C, 3D). The thickness of the DDF tendon is uniform as it moves over the metacarpophalangeal/metatarsophalangeal joint and through the manica flexoria (Figs. 3D, 7C). The SDF inserts on the proximal flexor surface of P2, and the DDF inserts on the flexor surface of P3.

### Arthrocentesis

The phalangeal joints were accessible via the dorsal arthrocentesis approach (Fig. 1D), however, it is important to note that in 20% of cases, we observed damage to the cartilage of the articular joint surfaces. During the dorsal approach, the needle injured the digital extensor tendons where it fuses with the metacarpophalangeal, metatarsophalangeal, and proximal interphalangeal joint capsule**.** It's worth mentioning that these observations were made during experiments conducted in a laboratory setting. We believe that when performing arthrocentesis on live animals in the field, the likelihood of such damage may be higher.

In this study, the lateral arthrocentesis approach to the proximal palmar/plantar pouches of the metacarpophalangeal/metatarsophalangeal and proximal interphalangeal joints was conducted under ultrasound guidance, as well as through a palpation-guided approach (Fig. 4C,D). No articular cartilage, tendons, nerves, or blood vessels were at risk of needle injury with this approach. Casting material injected via the lateral arthrocentesis approach successfully filled the relevant joint cavity and dorsal recess (Fig. 4C,D).

A palmar/plantar arthrocentesis approach is not recommended because of the location of the IOM, DDF, and SDF, as well as the presence of the footpad, in the case of the proximal and distal interphalangeal joints (Fig. 2C).

### Ultrasonography

The ultrasonographic images revealed that the tendons and other soft tissues surrounding the joint cavities appeared hyperechoic. In contrast, the synovial fluid within the joint cavities demonstrated hypoechoic characteristics. The digit joints were examined with ultrasonography from a dorsal, lateral, and palmar/plantar aspect, in an extended position (Figs. 3C, 5C–E, 6C,D, 7C). The epiphyseal lines of the distal ends of the metacarpal and metatarsal bones and the proximal ends of P1 and P2 were distinguished (Figs. 3C, 5C,D, 6C,D). Furthermore, the joint cavities of the metacarpophalangeal, metatarsophalangeal, proximal interphalangeal, and distal interphalangeal joints were identified via the dorsal aspect (Fig. 5C–E). P3 could not be viewed via the dorsal aspect because of the obstruction of the hoof capsule.

On the lateral aspect of the digit, our observations included the distal end of the metacarpal/metatarsal bones, the metacarpophalangeal/metatarsophalangeal joint cavities, P1, proximal interphalangeal joint cavities, and P2 (Fig. 6C,D). Furthermore, the large palmar/plantar pouches of the metacarpophalangeal/metatarsophalangeal and proximal interphalangeal joints were identified (Fig. 6C,D).

Palmar/plantar, the distal ends of the metacarpal/metatarsal bones, the IOM, SDF, DDF, the manica flexoria, the large metacarpophalangeal/metatarsophalangeal pouches, the axial and abaxial proximal sesamoid bones, the proximal end of P1, and the small pouch extending distally between the two proximal sesamoid bones were identified (Figs. 3C, 7C). The insertion of the IOM on the proximal sesamoid bones could be viewed under ultrasound guidance (Figs. 3C, 7C). The DDF and SDF were clearly seen on ultrasound imaging (Figs. 3C, 7C). Figure 7C indicates the thinning of the SDF as it moves over the proximal sesamoid bones and forms the manica flexoria. The proximal and distal interphalangeal joints and the distal ends of the P1, P2, and P3 could not be viewed via a palmar/plantar aspect because of the presence of the footpad.

### Computed tomography

The CT scans in this study provided three-dimensional images with exceptional detail, allowing for a clear visualization of the distal limb including joint cavities, recesses, and pouches of the metacarpophalangeal/metatarsophalangeal joints, as well as the proximal and distal interphalangeal joints (Figs. 3A,B, 5A,B, 6A,B, 7A,B). The CT results were in accordance with the ultrasound findings, supporting the accuracy of our anatomical observations.

## Discussion

In this study, our primary objective was to provide a detailed topographic anatomy of the phalangeal joints in the camel, laying the groundwork for precise landmarks essential for arthrocentesis procedures. Utilizing dissection, CT and ultrasound imaging methods, we meticulously examined the phalangeal joints’ cavities, recesses, and pouches. While we did not conduct quantitative measurements, our visual evaluations based on CT and ultrasound imaging did not show any appreciable topographical or anatomical differences between the joint cavities, joint recesses, or joint pouches between digits III and IV of the same limbs or between the left and right limbs. Our findings are consistent with previous reports by Nourinezhad et al.^29^, who similarly noted no significant differences between the phalanx measurements of digits III and IV in the same limb or between the right and left limbs, albeit with quantitative data indicating differences in phalanx length and width between forelimbs and hindlimbs.

Interestingly, the IOM completely inserts on the proximal sesamoid bones and does not extend a dorsal branch to fuse with the extensor tendons in dromedaries, in contrast to what is found in equines and bovines^30–32^. This unique anatomical adaptation of dromedaries allows the metacarpophalangeal/metatarsophalangeal joints to to flex and extend more than in equids during their unique biomechanical actions, especially during the movement associated with standing up. Another finding unique to dromedaries compared to bovines is that there is no communication between the metacarpophalangeal/metatarsophalangeal joint cavities of digits III and IV^23,33^. Thus, during arthrocentesis the metacarpophalangeal/metatarsophalangeal joints of digits III and IV must be injected separately.

Intriguingly, all casted samples showed a short axial and abaxial recess extending distally on P2. To the best of our knowledge, this axial and abaxial joint recess has not been reported in previously published literature. The assumption is that these joint recesses facilitate the slight adduction and abduction movement of the proximal interphalangeal joint.

The traditional arthrocentesis approach to the phalangeal joints of the dromedary is a dorsal one. This is an easily accessible approach^14,23^. However, caution must be exercised when using this approach, because the needle passing through the extensor retinaculum and tendons fused with the respective joint capsules^12^ and inevitably causes damage to the tendon structures. Furthermore, the flexion of joints, especially in the case of the metacarpophalangeal/metatarsophalangeal joint which allows for an extended range of flexion, expose the articular cartilage to potential needle injury. The current study investigated and evaluated a lateral approach to the proximal palmar/plantar pouches of the metacarpophalangeal/metatarsophalangeal and proximal interphalangeal joints as an alternative for the commonly used dorsal approach. The lateral approach to the metacarpophalangeal/metatarsophalangeal joint pouches is frequently used in horses and donkeys^31^. However, the exploration of this approach in dromedaries has not been extensively developed beyond it being mentioned as a potentially accessible injection approach by Smuts and Bezendenhout^8^. According to our research, no published study has investigated the feasibility of the lateral approach to the palmar/plantar proximal joint recesses of the proximal interphalangeal joint in dromedaries, nor has any guidance to such an approach been mentioned in publications.

The large proximal palmar/plantar pouches of both the metacarpophalangeal/metatarsophalangeal and the proximal interphalangeal joints can be injected under US guidance. Clearly, recognizable anatomical landmarks are critical during ultrasonographic imaging^21^. Thus, the distal end of the metacarpal/metatarsal bones, the proximal sesamoid bones, P1, and P2, as well the IOM, DDF, and SDF, were all used as landmarks when using the US guided arthrocentesis approach. The ultrasound needle-guided approach can be used to overcome the limitations experienced by clinicians using a palpation-guided approach, as discussed by Al-Sobayil et al.^12^.

In the absence of a ultrasound device, experienced clinicians can use a palpation-guided approach when injecting the proximal palmar/plantar pouches of the metacarpophalangeal/metatarsophalangeal and proximal interphalangeal joints. The distal ends of the metacarpal/metatarsal bones, IOM, and abaxial proximal sesamoid bones act as palpation landmarks for the lateral arthrocentesis approach. During extension of the MTCP joint, an easily palpable depression is formed in the area where the metacarpal/metatarsal bones, the IOM, and the abaxial proximal sesamoid bone meet. It is recommendable to insert the needle next to the abaxial proximal sesamoid bone, distally oriented toward the metacarpal/metatarsal bones to ensure that the needle successfully enters the palmar/plantar proximal pouches of the metacarpophalangeal/metatarsophalangeal joints. During extension of the MTCP joint, a depression between the abaxial articular prominence of P1 and the SDF can be palpated distal to the manica flexoria. The needle can successfully enter the palmar/plantar proximal pouch of the proximal interphalangeal joint when it is inserted in a distally oriented fashion as close to P1 as possible. The lateral approach, as concluded from our observations and the inherent topographical anatomy of the structures in this region, presents no evident risk of needle injury to tendons, nerves, veins, arteries, or joint cartilage. However, the lateral approach is not applicable to the distal interphalangeal joint because of the positioning of the hoof capsule and footpad. No palmar/plantar arthrocentesis approach is feasible for any of the phalangeal joints because of the flexor tendons, as well as the footpad in the case of the proximal and distal interphalangeal joints.

Given that all the distal forelimbs and hindlimbs utilized in this study were collected from freshly slaughtered camels, it is reasonable to consider that the arthrocentesis techniques described in this study may have applicability in living camels. However, it is essential to note that this conclusion primarily pertains to the lateral approach and that further in vivo investigations specifically evaluating the lateral approach are warranted to provide more definitive evidence.

## Conclusion

Because of the morphological and anatomical differences between horses, bovines, and dromedaries, it is critical that accurate and safe arthrocentesis techniques are developed specifically for dromedaries. The combination of CT, ultrasonography, casting, and dissection offered a uniquely comprehensive representation of the phalangeal joints of the dromedary.

When administering injections into the phalangeal joints of the dromedary, it is noteworthy that the lateral approach to the palmar/plantar proximal pouches of the metacarpophalangeal/metatarsophalangeal joints and the proximal interphalangeal joints may provide certain safety advantages. This approach is associated with a reduced risk of injury to the articulating cartilage surface. However, this approach is not feasible for the distal interphalangeal joints.

## Data Availability

The datasets generated during the current study are available from the corresponding author on reasonable request.
